# The Impact of Cultural Capital on Vaccine Attitudes among the Slovenian Public

**DOI:** 10.3390/vaccines10111947

**Published:** 2022-11-17

**Authors:** Andrej Kirbiš

**Affiliations:** Department of Sociology, Faculty of Arts, University of Maribor, 2000 Maribor, Slovenia; andrej.kirbis@um.si

**Keywords:** cultural capital, vaccine attitudes, vaccine hesitancy, education, cultural participation

## Abstract

Education and highbrow cultural participation—two dimensions of cultural capital—have previously been identified as determinants of vaccine attitudes, though the links have been mainly inconsistent across studies. The present quantitative study aimed to examine the effects of two dimensions of cultural capital (institutionalized and embodied cultural capital) on the pro-vaccine attitudes of the Slovenian public. A cross-sectional quantitative study was performed in November 2019, a few months prior to the COVID-19 pandemic. The non-probability sample survey was collected by inviting respondents over the age of 18 to participate using the snowball technique via e-mail, digital social networks (Facebook, Twitter and Instagram) and University of Maribor social network profiles. The sample was obtained through an online survey tool 1ka.si (N = 661; M_age_ = 34.9 years). The impact of education and highbrow cultural participation on vaccine attitudes was examined, controlling for sociodemographic variables (gender, age and size of residential settlement) and economic variables (income and family economic status) in multivariate analyses. Bivariate analyses indicated that pro-vaccine attitudes were significantly more likely to be expressed by men, younger respondents, those with lower incomes, higher perceived family economic status, living in urban areas and by those who are more frequently engaged in highbrow cultural activities, while education had no impact on vaccine attitudes. The results did not substantially change when multiple regression models were employed. Our study indicated that cultural capital has an inconsistent impact on vaccine attitudes; while education has no impact, highbrow cultural participation increases pro-vaccine attitudes. The results suggest a multi-type approach is needed to address vaccine scepticism among the Slovenian public.

## 1. Introduction

Slovenia has a comparatively low uptake of vaccines for vaccine-preventable diseases, including seasonal influenza [1], human papillomavirus [2,3] and rotavirus [4], while vaccine hesitancy in the Slovenian public is among the highest in the world [5]. A study of Slovenian mothers with young children found that just over half of mothers expressed intention to vaccinate their children in the case of non-mandatory vaccination [6]. Other Slovenian subgroups and the general public also show relatively high scepticism of vaccines [7].

Vaccine uptake largely depends on understanding the social determinants of vaccine attitudes among the general public [8,9,10]. Research indicates the resources, or “capital”, people have at their disposal have a significant impact on their health behaviours and outcomes [11,12,13], including vaccine uptake and attitudes [14,15,16]. French sociologist Pierre Bourdieu referred to capital as “all the goods, material and symbolic … that present themselves as rare and worthy of being sought after” in a particular society [17]. Much of the previous research has indicated that economic capital, such as income and wealth [16,18,19], and social capital, such as social connections and support [20,21,22,23], influence vaccine uptake and vaccine attitudes.

However, only a few studies have examined the role of cultural capital and its various dimensions in vaccine attitudes. In the next section, we briefly discuss the results of previous studies on the link between cultural capital and vaccine attitudes.

### Cultural Capital and Vaccine Attitudes

*Cultural* capital, which can be regarded as familiarity with the dominant cultural codes within a society and the symbolic and informational resources for action, which are the results of one’s position in the social structure [24,25,26,27], has also emerged as an important determinant of vaccine attitudes and uptake. Two forms of cultural capital have been previously examined, although to an unequal extent, yet both were found to impact the public's perceptions of vaccines. First, education, a form of institutionalized cultural capital, e.g., educational degrees and professional titles [28,29], has been among the most commonly analysed determinants of vaccine attitudes (usually included in socioeconomic models), yet results have largely been inconsistent across various examined cultural contexts. Some studies suggest a higher educational level increases positive vaccine attitudes [5,30,31,32,33], and more-educated parents were found to have higher trust in vaccine safety [34,35].

Some studies, on the other hand, show that higher educational levels are associated with negative vaccine attitudes [10,36,37]. An analysis of eleven European countries, for example, showed that in several countries, people with higher education expressed more negative attitudes toward vaccines [38]. Overall, in the regions of Eastern Europe, Central Africa and South Africa, negative attitudes are more likely to be expressed by individuals with higher education [39]. Finally, some studies in developed countries suggest that vaccine hesitancy is not statistically significantly associated with educational attainment [40,41,42], including in Slovenia [1,2,6].

Besides education, there is some evidence that another cultural capital dimension, embodied or incorporated cultural capital, which involves lasting, legitimate cultural dispositions, tastes and behaviours that are internalized during the socialization process [27,43,44,45], may also play a role in vaccine attitudes and uptake. For example, qualitative empirical research indicates that vaccine hesitancy may be more pronounced among those with higher cultural capital [14,22]. Attwell and colleagues, for example, emphasize that anti-vaccine practices “are created and promoted by an […] elite […] who has the power to define non-vaccination as desirable—a form of distinction.” [23].

We argue that the inconsistent impact of education on vaccine attitudes across previous studies could partly be due to the unobserved variance in embodied cultural capital such as highbrow cultural participation, which has not yet been included in quantitative analyses on vaccine attitudes. Highbrow cultural activities (e.g., attendance of theatre, concerts of classical music, opera, visiting museums or cultural sites) are regarded as “the most general form of prestigious culture in the West, and thus a privileged indicator of cultural capital” [46,47] and have been a widely used indicator of social distinction within cultural capital and health literature [48,49]. The influential paper by Abel [44] conceptualised the role of cultural capital and its impact on health. Abel argues that health-relevant cultural capital comprises “culture-based resources that are available to people for acting in favour of their health” and includes “health-related values, behavioural norms, knowledge and operational skills.” In this sense, attitudes toward vaccines can be regarded as embodied cultural resources where, within a range of choices provided by economic and social capital, vaccine-related perceptions and behaviours are formed and enacted [44].

There is convincing evidence that in Slovenia, cultural capital significantly impacts health [43]. However, in recent years, scepticism and rejection of vaccines are regarded as a form of symbolic capital, and more educated groups, who are at the same time also more likely to be culturally engaged, may use vaccine scepticism as a marker of distinction [23]. Slovenia is a Central-European country that is ranked among the most developed on the Human Development Index (ranked 23rd) with a relatively high GDP per capita, mean education levels and life expectancy [50]. Along with other European, post-communist democracies, it is regarded as a younger but stable democracy. For example, Freed House [51] categorizes Slovenia as a “free” and “consolidated” democracy, ranked similarly to other established Western democracies. Regarding vaccine policies in Slovenia, there is compulsory vaccination against some vaccine-preventable diseases, including Haemophilus influenzae type B, DTP (diphtheria, tetanus, pertussis) and MMR (measles, mumps, rubella). MMR vaccination is obligatory for children when entering public or private kindergarten [52]. When it comes to parental decision-making on the vaccination of their children, doctors are regarded as the most-trusted source of information on vaccines and vaccination, similar to other countries [53].

Since there is a lack of studies among the Slovenian public on vaccine attitudes [54], we built on the insights of previous research and, in the present study, examined the role of both education and highbrow cultural participation on general vaccine attitudes. To the best of our knowledge, their impact has not yet been compared in quantitative studies within the public health literature. We hypothesized that (H1) education is significantly linked to pro-vaccine attitudes; (H2) highbrow cultural activities, due to their function of distinction, have a negative impact on pro-vaccine attitudes, as suggested by the above-mentioned qualitative studies; and (H3) the impact of education on pro-vaccine attitudes decreases when simultaneously controlling for highbrow culture.

## 2. Methods

### 2.1. Sample

A cross-sectional quantitative study was performed in November 2019, a few months before the COVID-19 pandemic. The non-probability sample survey was collected by inviting respondents over the age of 18 to participate using the snowball technique [55] via e-mail, digital social networks (Facebook, Twitter and Instagram) and University of Maribor social network profiles. The sample was obtained through an online survey tool 1ka.si, and it comprised 661 Slovenians (M_age_ = 34.9 years). The only inclusion criterion was being 18 years of age or older. After reading the written consent form and explicitly agreeing to take part in the study and to the publication of the results, participants were asked to complete the survey reflecting on both their attitudes and behaviours regarding vaccination and to provide their sociodemographic information.

### 2.2. Measures

Vaccine attitudes were measured with four items. Three items were previously used in a cross-national study by Larson and colleagues (5) (1 = strongly disagree; 2 = disagree; 3 = neither; 4 = agree; 5 = strongly agree): “In general, I think vaccines are effective.”, “In general, I think vaccines are safe.”, “Vaccines are important for a child’s health.”. We also used an additional item “People who do not vaccinate their children are endangering others.”. We created a four-item summation variable with higher scores indicating pro-vaccine attitudes (Cronbach’s Alpha = 0.98).

Institutionalized cultural capital was measured on an 11-point scale as the highest currently acquired educational level. We recoded the values into a 3-point scale (1 = secondary education or less; 2 = post-secondary education; 3 = Master’s degree or Ph.D.).

Embodied cultural capital was measured with the following question on highbrow cultural participation: “How often in the last 12 months did you do the following activities in your free time?” (1 = never; 6 = every day). The items measured were as follows: visited “art gallery, museum”, “theatre”, “opera, ballet, classical concert”, “historic sites, places” and “read a book”. The items were summated into a scale (Cronbach’s Alpha = 0.75). Due to the skewness of the summation scale, quartile groups were calculated and used in multivariate analyses.

We used five control variables in our multivariate models: gender (0 = male; 1 = female), age (in years) and size of residential settlement (1 = less than 2000 residents; 2 = 2000 to 50,000 residents; 3 = more than 50,000 residents). Personal income was stated by respondents (in EUR). Finally, respondents rated their family's economic status on a scale from (1 = strongly below average) to 10 (highly above average).

### 2.3. Plan of Analysis

Statistical Package for the Social Sciences Program (IBM SPSS Statistics Versions 27) was used for the analyses. First, descriptive statistics were examined. Second, bivariate correlations were calculated to test associations between control variables, institutionalized and embodied cultural capital and pro-vaccine attitudes. Third, pro-vaccine attitudes were analysed within three linear regression models. In the final regression model, we examined the predictive value of both cultural capital measures, adjusted for sex, age, residential settlement, income and family economic status.

## 3. Results

### 3.1. Sample Characteristics

In total, 661 respondents took part in the study. The median age was 34.92 years; 76.6% were women. Table 1 presents the sociodemographic characteristics of the respondents as well as their cultural capital and vaccine attitudes.

Approximately an equal proportion of respondents lived in rural areas and urban areas. A majority of respondents had an income between the minimum and average income in Slovenia. In addition, almost half of the respondents assessed their family's economic status as average.

Regarding cultural capital, post-secondary education was attained by three out of five respondents. Analysis of cultural participation indicated that reading books, visiting historic sites and visiting art galleries and museums were the most frequent while visiting theatre and opera, ballet and concerts of classical music were the least frequent among survey respondents.

Finally, just over half of respondents expressed pro-vaccine attitudes on all four vaccine measures, indicating that respondents were relatively sceptic of vaccines.

### 3.2. Bivariate Associations between Cultural Capital and Vaccine Attitudes

Table 2 shows the results of bivariate analyses between variables of interest. Women are less likely to express pro-vaccine attitudes (r = −0.26; *p* < 0.001), as are older respondents (r = −0.11; *p* < 0.01). Those who live in larger, urban residential settlements are more likely to express pro-vaccine attitudes (r = 0.19; *p* < 0.001). Turning to economic capital, income (r = −0.07; *p* > 0.05) and family economic status (r = 0.05; *p* > 0.05) were not found to be significantly associated with vaccine attitudes. Finally, respondents with more frequent highbrow cultural participation are more likely to express pro-vaccine attitudes (r = 0.09; *p* < 0.05), while there was no statistically significant impact of education on vaccine attitudes in bivariate analysis (r = −0.01; *p* > 0.05).

### 3.3. Multivariate Analysis

Table 3 presents the results of three multiple linear regression models. Model 1 included sociodemographic control variables (gender, age and size of residential settlement) and two economic variables (income and family economic status). All sociodemographic and economic variables proved to be statistically significant predictors of pro-vaccine attitudes in Model 1. Among sociodemographic controls, women were less likely to express positive vaccine attitudes (β = −0.32; *p* < 0.001), as were older respondents (β = −0.17; *p* < 0.001) and those who live in smaller residential settlements (β = 0.18; *p* < 0.001). Among economic controls, lower income (β = −0.16; *p* < 0.01) but higher perceived family economic status (β = 0.10; *p* < 0.05) proved to be predictors of pro-vaccine attitudes. Model 2 included sociodemographic and economic controls and education, the first of the two cultural capital dimensions. All sociodemographic controls and economic controls remained significant predictors of vaccine attitudes, while there was no statistically significant impact of education (β = 0.01; *p* > 0.05). Finally, Model 3 included all previous predictors and highbrow cultural participation. All variables included in the previous two models mainly had the same and significant impact in Model 3, while cultural participation also turned out be a significant predictor of pro-vaccine attitudes (β = 0.09; *p* < 0.05). Based on these results, we can conclude that negative vaccine attitudes are more likely expressed—in order of the predictive strength—by women, older individuals, those who live in smaller residential settlements, have higher income but lower perceived family economic status and those who are less frequently participating in highbrow cultural activities. In addition, we found that the explained variances were relatively low in all three models (R^2^ ranging from 14.4% to 15.1%), indicating that other unobserved variables may impact vaccination attitudes among the Slovenian public. 

## 4. Discussion

Our study examined the role cultural capital plays in impacting pro-vaccine attitudes, controlling for sociodemographic and economic determinants. Regarding the examined control variables, our findings suggest that men are more likely to express pro-vaccine attitudes than women. This is consistent with a study conducted in Canada [33], although some previous studies also found women are more likely to express pro-vaccine attitudes and behaviours [56,57]. It seems then that among the Slovenian public, women are more vaccine-sceptical than men. Similar results were also reported for COVID-19 vaccine hesitancy in Slovenia [58] and other countries [59]. Women’s greater scepticism towards vaccines may stem from their maternal roles, as they are disproportionately responsible for healthcare decisions concerning their children [60], including vaccine decisions. In addition, the majority of the anti-vaccination movement is represented by women, with the discourse centred around distrust in government, media and conspiracy beliefs [61]. However, maternal opposition to vaccines is often rooted in “strong beliefs regarding health, diseases, and prevention that could be labelled “holistic”, “natural,” or “alternative.” [62].

In addition, we found younger individuals are more likely to express positive attitudes toward vaccination, in line with some earlier research [63]; however, previous studies’ results are, again, mixed [5]. In addition, some Slovenian data suggest that the role of age in vaccine hesitancy may not be linear. For example, COVID-19 vaccine hesitancy was found to be highest among the middle-aged group (30–39-year-olds), while 18–29-year-olds proved to be the least COVID-19 vaccine-hesitant [58], echoing the results of the present study. Further studies are needed, as the impact of age may depend on the type of vaccine. Similar to earlier research in Europe [38], Slovenian respondents living in urban areas express more favourable attitudes toward vaccines.

Interestingly, we found that lower self-assessed family economic status showed a negative impact on vaccine attitudes, while those with lower income expressed more favourable vaccine attitudes, although earlier research is, again, inconsistent [19,64]. It seems that *objective* and *subjective* economic capital may differ in terms of their roles in forming vaccine attitudes. Having more objective resources, including income but, for example, also time, could also mean more resources to gather health- and vaccine-related information (and misinformation), to search for information online, including reading conspiracy-related social media websites, and to customize one’s own and family healthcare choices [65]. In addition, those with higher levels of income, informational resources and fewer time obligations are “best situated to manage illness, lengthy quarantines, or missed work opportunities that might result from exposure to vaccine-preventable diseases than are families with fewer resources.” [14]. 

We hypothesized (H1) that education would be significantly linked to pro-vaccine attitudes, which was not confirmed by our data. It seems that education has no impact on vaccine attitudes, as indicated by some previous studies [40,41], including in Slovenia [1,2]. H2 predicted highbrow cultural activities, due to their function of social distinction, would have a negative impact on pro-vaccine attitudes, yet the opposite seems to hold for the Slovenian public. Our results suggest that highbrow cultural participation is an important positive resource (capital) that increases pro-vaccine attitudes, which in turn increases vaccine uptake [8,9]. Our study suggests that in Slovenia, cultural resources may impact health behaviours (vaccine uptake) and health outcomes (immunity against vaccine-preventable diseases) through health-related attitudes (e.g., favourable vaccine attitudes or health-related cultural capital) [44]. Finally, we predicted that the impact of education on pro-vaccine attitudes would decrease when simultaneously controlled for highbrow culture (H3). The insignificant impact of education on vaccine attitudes did not change when cultural participation was included in the final regression model.

One possible reason for the lack of educational impact on vaccine attitudes might be the egalitarian historical and cultural context. For example, Slovenia has the second-lowest income inequality in the world [50], while the cultural orientations of Slovenians are likewise strongly pro-egalitarian [66]. Hjellbrekke and colleagues argue that in egalitarian, socially democratic and welfare countries, there is still a marked social distribution of lifestyles, especially in cultural knowledge, participation and taste. In fact, “egalitarian values can be compatible with quite severe social hierarchies, and an ‘egalitarian ideology’ can conceal, and even help to maintain, the hierarchical structures of society.” [67]. In post-modern societies, the expansion of tertiary education has decreased its social status, while other forms of cultural capital may function as a more potent marker of distinction. The results of our study suggest that in Slovenia, it is not education but embodied cultural capital—in the form of highbrow cultural participation—that functions as a marker of distinction in vaccine attitudes. This means that cultural participation’s impact on vaccine attitudes is not in the direction previous qualitative studies from other countries suggest—highbrow cultural engagement in Slovenia increases positive health-related (i.e., pro-vaccine) attitudes. However, our results are similar to other quantitative studies indicating that cultural participation plays a vital role in health-related attitudes and behaviours. Future studies should examine quantitative and qualitative data from the same country to comprehensively test cultural participation’s role in vaccination attitudes and behaviour.

Our study suggests that highbrow cultural participation increases pro-vaccination attitudes, which may, in turn, increase health inequalities among Slovenian adults since cultural participation has consistently been found unequally distributed across social strata [68,69,70], including in Slovenia [43]. While our findings are not in line with qualitative studies in other countries, e.g., in Australia, diverging results could be explained by focusing on “the place’ of vaccine rejection [which] needs to be understood as a reified or valorized practice within some social groups, but not necessarily others” [23]. It seems that in the Slovenian context, cultural capital functions more in line with the traditional understanding of cultural capital and empirical findings on its impact [24,44,71], whereby it is conceptualized as culture-based resources that people can employ in favour of their health, including health-related values and attitudes, as confirmed in previous quantitative empirical analyses.

Our results also suggest the Slovenian public is not a typical case where a lack of personal or familial resources decreases favourable vaccine-related attitudes across the board; in fact, different types of resources seem to have different impacts on vaccine attitudes. Among economic resources, higher income decreases while subjective family economic status increases pro-vaccine attitudes. Similarly, highbrow cultural resources increase positive vaccine attitudes, while education has no impact. Our results indicate the need for tailored public health campaigns focusing especially on women (including mothers), high-incomers and populations from rural Slovenian areas but also those who are less culturally engaged.

Despite our study presenting evidence of the impact cultural capital has on vaccine attitudes, several study limitations need to be mentioned. Our study was cross-sectional, which precluded us from inferring causation. Secondly, the employed survey sample was not nationally representative, so our results need to be further tested in representative studies. Thirdly, our models had relatively low predictive power since the list of our control and predictor variables was not exhaustive; future studies should, therefore, also control for the impact of other cofounders of vaccine attitudes previously found in the literature, including beliefs in alternative medicine [72,73], political orientations [74,75] and beliefs in conspiracy theories [76,77]. However, the main aim of our study was not to construct models that would necessarily explain a large amount of variance in pro-vaccination attitudes but to examine whether the two cultural capital variables have an impact on vaccination attitudes, regardless of the size and controlling for the impact of other predictors. Finally, we examined two dimensions of cultural capital (institutional and embodied capital), while a third dimension (objectified cultural capital) may also prove worthwhile to examine (e.g., possession of valued cultural goods in the household, such as the number of books, musical instruments, artwork). In addition, other study methods of identifying cultural activity could also be examined, including different profiles of cultural consumers and their vaccine attitudes. Future research should aim to overcome these limitations.

## 5. Conclusions

Our cross-sectional study indicates that in our sample of Slovenians, pro-vaccine attitudes are more likely to be expressed by men, younger individuals and those living in urban areas. Higher income has a negative impact on pro-vaccine attitudes, perceived family economic status has a positive impact, while cultural capital similarly plays an inconsistent role: institutional cultural capital (education) is not a significant predictor of vaccine attitudes; in contrast, more frequent highbrow cultural participants are more likely to express positive vaccine attitudes. Our findings suggest that public health professionals and vaccine campaigns in Slovenia should, among others, also focus on those segments of the general public that are disengaged from highbrow cultural activities. At the same time, higher-income groups also need to be addressed, as do people from all levels of educational backgrounds and, most importantly, the female population. The inconsistent impact of economic, educational and highbrow cultural resources suggests a multi-type approach is needed to address vaccine scepticism among the Slovenian general public, including in the aftermath of the COVID-19 pandemic.

## Figures and Tables

**Table 1:** Descriptive statistics of vaccine attitudes, cultural capital and control variables.

	Sociodemographic Feature	n	%
Gender (female)	Female	506	76.6
	Male	155	23.4
Age	18–29 years	218	33.0
	30–39 years	244	36.8
	40+ years	199	30.2
Size of residential settlement	Less than 2000 residents	240	36.3
	2000 to 50,000 residents	182	27.5
	More than 50,000 residents	239	36.2
Income	Up to 850 EUR monthly	189	28.6
	851–1300 EUR monthly	264	39.9
	More than 1300 EUR monthly	208	31.5
Family economic status	1–4 (below average)	98	14.9
	5–6 (average)	313	47.5
	7–10 (above average)	248	37.6
Education	Secondary education or less	183	27.7
	Post-secondary education	394	59.6
	Master’s degree or PhD	84	12.7
Cultural participation *	Art gallery, museum	324	49.0
	Theatre	261	39.5
	Opera, ballet, classical concert	182	27.6
	Historic sites, places	498	75.5
	Read a book	581	87.9
Vaccine attitudes **	In general, I think vaccines are effective.	376	56.9
	In general, I think vaccines are safe.	337	51.0
	Vaccines are important for a child’s health.	0.354	53.6
	People who do not vaccinate their children are endangering others.	339	51.3

Note. * Visited at least a few times a year or more frequently; ** Agree or strongly agree with the statement.

**Table 2:** Bivariate correlations between pro-vaccine attitudes, two cultural capital dimensions and control variables.

	M/%	SD	Min./Max.	1.	2.	3.	4.	5.	6.	7.
1. Pro-vaccine attitudes	3.13	1.61	1–5	-						
2. Gender (female)	76.6	/	/	−0.26 ***	-					
3. Age	34.92	9.99	18–72	−0.11 **	−0.27 **	-				
4. Size of residential settlement	2.00	0.85	1–3	0.19 ***	−0.06	0.10 **	-			
5. Income	1292	925	0–5000	−0.07	−0.25 **	0.32 ***	0.09 *	-		
6. Family economic status	5.93	1.62	1–10	0.05	−0.09 *	0.12 **	0.13 ***	0.46 ***	-	
7. Education	1.85	0.62	1–3	−0.01	−0.10 *	0.35 ***	0.16 ***	0.25 ***	0.21 ***	-
8. Cultural capital	2.70	0.71	1–6	0.09 *	−0.01	0.13 ***	0.14 ***	0.11 **	0.10**	0.29 ***

Note. * *p* < 0.05; ** *p* < 0.01; *** *p* < 0.001.

**Table 3:** Multiple linear regression of pro-vaccine attitudes.

	Model 1 (R^2^ = 0.145; *p* < 0.001)		Model 2 (R^2^ = 0.144; *p* < 0.001)		Model 3 (R^2^ = 0.151; *p* < 0.001)	
	B (SE)	β	B (SE)	β	B (SE)	β
Gender (female)	−1.22 (0.14)	−0.32 **	−1.22 (0.14)	−0.32 **	−1.23 (0.14)	−0.33 **
Age	−0.34 (0.08)	−0.17 **	−0.34 (0.08)	−0.17 **	−0.35 (0.08)	−0.17 **
Size of residential settlement	0.35 (0.07)	0.18 **	0.35 (0.07)	0.18 **	0.33 (0.07)	0.17 **
Income	0.00 (0.00)	−0.16 **	0.00 (0.00)	−0.16 **	0.00 (0.00)	−0.16 **
Family economic status	0.09 (0.04)	0.10 *	0.09 (0.04)	0.09 *	0.09 (0.04)	0.09 *
Education			0.03 (0.10)	0.01	−0.04 (0.11)	−0.01
Cultural participation					0.13 (0.05)	0.09 *

Note: * *p* < 0.05; ** *p* < 0.001.

## Data Availability

The data are available upon reasonable request to the author.
